# Evaluation of the health promotion effect of hepatitis B prevention and treatment in the Zhejiang demonstration area, China

**DOI:** 10.1186/s12889-022-14540-8

**Published:** 2022-11-14

**Authors:** Hongli Qin, Yan Qiu, Meike Ying, Jingjing Ren

**Affiliations:** Department of General Practice, First Affiliated Hospital, Zhejiang University School of Medicine, Hangzhou, 310003 China

**Keywords:** Health literacy, Hepatitis B prevention and control, Health education, “Internet +”

## Abstract

**Background:**

To investigate the health literacy level and health promotion effect of hepatitis B prevention and treatment in the demonstration area of Zhejiang Province.

**Methods:**

The National Science and Technology Major Health Education Group took 6 demonstration areas in Zhejiang Province as the whole research site. After the sample size (*N*=2160 people) was determined, a multistage stratified cluster sampling method was used to conduct a questionnaire survey in 2018 (before health education) and 2019 (after health education). Stata 12 statistical software was used to analyse the status and improvement rate of hepatitis B health literacy of residents in the demonstration area before and after health education and compare the health promotion effects of different health intervention methods.

**Results:**

Before and after health education, there was no significant difference in the basic information of the subjects (*P*>0.05). After the health education intervention, the level of hepatitis B health literacy of residents in the demonstration area increased by 11.8%, and the difference was statistically significant (*P* < 0.001). The awareness rate of hepatitis B transmission was low before health education but increased after health education. The form of "Internet +" health education may better improve the residents' health literacy level about hepatitis B prevention and treatment.

**Conclusion:**

After health education, the level of health literacy of residents in the Zhejiang demonstration area about hepatitis B prevention and control significantly improved, but there is room for further improvement. In the future, targeted health education intervention should be carried out, and the health education mode of "Internet +" can achieve better results to effectively prevent and control hepatitis B.

The large-scale field epidemiology and _intervention_ study on the prevention and treatment of AIDS, viral hepatitis, tuberculosis and other major infectious diseases in Zhejiang Province is the pilot site of the national science and technology major project to reduce the morbidity and mortality of AIDS, viral hepatitis, tuberculosis and other major infectious diseases (referred to as "three diseases and two rates"). During the 12th Five-Year Plan Period (2011~2015), with reference to no. 3 of the announcement from the Ministry of Health regarding the health literacy, basic knowledge and skills (try out) of Chinese citizens [1], the group has developed a set of community residents, such as the hepatitis B prevention and treatment of infectious diseases health literacy assessment system, and uses this system to evaluate the community crowd health literacy baseline of three infectious diseases. During the 13th Five-Year Plan period (2016~2020) [http://www.nhc.gov.cn/qjjys/s3593k/201611/9db052e9254c4e2c9546dda3ecf125a3s.html], based on traditional health education, we made full use of the internet and other new media of healthy education to perform health education activities. All-round, three-dimensional, multi-form and systematic health education on the prevention and treatment of infectious diseases was conducted by community General Practitioners (GPs) to optimize the comprehensive model of health education on major infectious diseases.

The concept of health literacy first appeared in the literature in 1974 [2] and was later introduced into China. It was initially applied to prevent and control chronic noncommunicable diseases and provide health education. Reaching a consensus on a definition of health literacy was complicated by the multiple skill categories and applications that were increasingly identified as necessary to be literate in relation to one’s health. In this study, we adopted the following definition: Health literacy refers to the ability of individuals to access and understand basic health information and services, and to use these information and services to make the right decisions to maintain and promote their own health. We evaluated it by KAB (knowledge-attitude-belief) and basic health skills. The proposal of health literacy provided a theoretical basis for the investigation of health literacy of hepatitis B [3].

Improving urban and rural residents' health literacy is conducive to improving people's ability to identify and solve their own health problems, which is one of the main indicators of the "Healthy China 2030" plan outline [4]. Health literacy is a stronger predictor of population health than socioeconomic factors, age, ethnicity and other factors [5]. In this paper, the investigation of hepatitis B-related health literacy in the Zhejiang demonstration area and the effect of health education intervention are reported.

## Research methods

### Study population

Inclusion criteria: Permanent residents aged 15-69 in demonstration areas of Zhejiang Province with certain learning ability, normal thinking and language expression ability; able to communicate orally and in writing; able to complete the assessment system or understand the questions of the community GP. Sample size calculation: *N*=400*q/p; q= 1-P, based on the national infectious disease prevention literacy in 2013 (*P* =17.12%); considering the 10% lost to follow-up rate (*N*=2130 people). After the sample size was determined, random sampling was used to select 6 demonstration areas with 355 people in each area, who were randomly divided into three groups with 118.33 people per group. The integer was 120 people, and the final number was 2160 people. Multi-stage unequal probability sampling was used.

### Specific implementation plan

The study was conducted in six districts in Zhejiang Province, China, from 1 March 2018 to 31 December 2020. There were two intervention groups. Group I would be given WeChat-based health education related to hepatitis B prevention and control (once or twice monthly) by clinicians from the Department of general practice in general hospitals .Group II would be given traditional plus WeChat-based health education by clinicians from the Department of general practice in general hospitals and general practitioners in the community. The control group was only given traditional health education (such as clinic education and pamphlet education) by general practitioners in the community,it was usually done during outpatient visits or hepatitis B vaccination clinics or on July 28-World Hepatitis Day. A total of 2160 participants were randomly divided into groups I and II and the control group in six districts. All participant’s intervention started as long as they were enrolled .Therefore, all the participant started with the intervention at the same time and completed the 1-year intervention. The research team conducted a baseline survey in March 2018 and a second survey in March 2019.

The questionnaire included the basic information of the respondents, the concept and basic knowledge of hepatitis B, the attitude and behavior of hepatitis B (namely, Knowledge-Attitude-Behavior) and health skills. For hepatitis B prevention and control concept and basic knowledge questions: "answer correctly" would be recorded as "1" , "answer wrong" would be recorded as "0" . For multiple choice questions, If the correct answer rate of all options was more than 60%, the question was judged to be correct and marked as 1 point. For attitude and behavior of hepatitis B: a good attitude and behavior was marked as "1", and "0" means none. A score of "1" was denoted with health skills, and a score of "0" was denoted without health skills. A score of 80% or more of the total score was considered to have health literacy of hepatitis B.

Investigators received unified training, and questionnaires were collected by special personnel. Unqualified questionnaires were discarded according to quality control requirements.

The observation index was the hepatitis B health literacy level of the community population in the demonstration area, and the evaluation index was the improvement rate of the hepatitis B health literacy level.

### Statistical analysis

Epidata was used to establish a database for double data entry,and unqualified questionnaires were eliminated. Stata 12 software was used for statistical analysis, the hepatitis B health literacy level of the subjects was expressed by frequency and percentage, Chi-square test was used for comparison between groups, *P*<0.05 (2-tailed test) was considered as statistically significant.

## Research results

### Basic information of survey subjects

As shown in Table 1, before health education, 2160 questionnaires were distributed, and 2033 were responsed, with a recovery rate of 94.1%. Among the 2,033 people surveyed, the male-to-female ratio was 1.09 to 1. Most of them were aged 25 to 55, which accounted for 85.44% of the sample. (Table 1).

**Table 1:** Demographic characteristics of the respondents (n,%)

Group	Before health education	After health education
Gender		
Male	1060 (52.14)	1028 (50.57)
Female	973 (47.86)	1005 (49.43)
Age (y)		
15~	296 (14.56)	298 (14.66)
25~	451 (22.18)	495 (24.35)
35~	403 (19.82)	407 (20.02)
45~	474 (23.32)	495 (24.35)
55~	409 (20.12)	338 (16.63)
Nationality		
Han	1995 (98.13)	2020 (99.36)
Minority	13 (0.64)	8 (0.39)
Else	25 (1.23)	5 (0.25)
Educational level		
Illiterate and semiliterate	48 (2.36)	44 (2.16)
Primary school	241 (11.85)	196 (9.64)
Junior high school	655 (32.22)	662 (32.56)
Senior high/technical school	523 (25.73)	549 (27.00)
Junior college/Bachelor degree or above	544 (26.76)	567 (27.89)
Unknown	22 (1.08)	15 (0.74)
Occupation		
Civil servants/employees of enterprises and public institutions	192 (9.44)	159 (7.82)
Professional and technical staff	222 (10.92)	229 (11.26)
Clerical and associated personnel	139 (6.84)	146 (7.18)
Business and service personnel	465 (22.87)	416 (20.46)
Agriculture, forestry, fishery or water industry personnel	223 (10.97)	222 (10.92)
Production and transportation workers	185 (9.10)	198 (9.74)
Else	607 (29.86)	663 (32.61)
Marital status		
Spinsterhood	430 (21.25)	439 (21.59)
Married	1528 (75.16)	1515 (74.52)
Widowed	19 (0.93)	19 (0.93)
Divorced	41 (2.02)	28 (1.38)
Unstated marital status	15 (0.74)	32 (1.57)
Income (yuan/month)		
<1000	294 (14.46)	270 (13.28)
1000~3000	641 (31.53)	527 (25.92)
3000~5000	884 (43.48)	949 (46.68)
5000~10000	187 (9.20)	261 (12.84)
≥10000	27 (1.33)	26 (1.28)
Payment method of medical expenses		
Basic medical insurance for urban workers	1024 (50.37)	981 (48.25)
Basic medical insurance for urban residents	414 (20.36)	381 (18.74)
New rural cooperative medical insurance	474 (23.32)	552 (27.15)
poverty relief	3 (0.15)	6 (0.30)
Commercial health insurance	14 (0.69)	7 (0.34)
Free medical care	21 (1.03)	15 (0.74)
Full medical care	47 (2.31)	37 (1.82)
Else	36 (1.77)	54 (2.66)

After health education, 2160 questionnaires were distributed, and 2142 were responsed, with a recovery rate of 99.17%. A total of 2033 questionnaires matched with those before health education were selected for analysis according to the object coding. (Table 1). Before and after health education, there was no significant difference in the basic information of the subjects (*P*>0.05).

### Health literacy

#### Analysis of hepatitis B health literacy of respondents with different characteristics before and after health education

After the health education intervention, the level of hepatitis B health literacy of residents in the demonstration area increased by 11.8%, and the difference was statistically significant (*P* < 0.001). Before and after health education, there was no significant difference in the health literacy level of hepatitis B prevention and treatment between genders. Both male and female residents' health literacy levels were significantly improved. Before health education, the health literacy level of residents' hepatitis B prevention and control basically showed a trend of decline with the increase of age, and the health literacy level was the highest in the age group of 35 ~ years old, and the lowest in the age group of 55 + years old. After health education, the level of health literacy of the elderly population over 55 years old still had a low level of health literacy. Before health education, the level of hepatitis B health literacy of residents with college, bachelor or above education level was the highest. The health literacy level of residents showed an increasing trend with the change of income, and the health literacy level of people with more than 10000 (yuan/month) of the income was the highest, reaching more than 80%.After health education, the level of health literacy of residents with primary school education or above was higher than that before (*P*<0.05). The health literacy level of residents<1000, 3000~5000 and 5000~10000 (yuan/month) of the income was significantly improved (*P*<0.05). See Table 2 for details.

**Table 2:** Comparison of the hepatitis B health literacy levels of respondents with different characteristics before and after health education

Group	Hepatitis B health literacy(n,%)			
	Before	After	*X*^*2*^	*P*
Gender				
Male	581 (54.81)	697 (67.80)	37.089	<0.001
Female	544 (55.91)	668 (66.47)	23.225	<0.001
Age(y)				
15~	151 (51.01)	221 (74.16)	33.995	<0.001
25~	314 (69.62)	385 (77.78)	8.134	0.004
35~	287 (71.22)	333 (81.82)	12.678	<0.001
45~	239 (50.42)	294 (59.39)	7.875	0.005
55~	134 (32.76 )	132 (39.05)	3.194	0.074
Nationality				
Han	1,105 (55.39)	1,361 (67.38)	60.871	<0.001
Minority	3 (23.08)	3 (37.50)	0.505	0.477
Else	17 (68.00)	1 (20.00)	4.000	0.046
Educational level				
Illiterate and semiliterate	12 (25.00 )	13 (29.55)	0.240	0.624
Primary school	57 (23.65)	69 (35.20)	7.031	0.008
Junior high school	318 (48.55)	386 (58.31)	12.602	<0.001
Senior high/technical school	296 (56.60)	382 (69.58)	19.426	<0.001
Junior college/Bachelor degree or above	433 (79.60)	505 (89.07)	18.937	<0.001
Unknown	9 (40.91)	10 (66.67)	2.369	0.124
Occupation				
Civil servants/employees of enterprises and public institutions	137 (71.35)	123 (77.36)	1.633	0.201
Professional and technical staff	161 (72.52)	202 (88.21)	17.663	<0.001
Clerical and associated personnel	103 (74.10)	116 (79.45)	1.146	0.284
Business and service personnel	243 (52.26)	286 (68.75)	24.892	<0.001
Agriculture, forestry, fishery or water industry personnel	87 (39.01)	91 (40.99)	0.181	0.670
Production and transportation workers	117 (63.24)	138 (69.70)	1.790	0.181
Else	277 (45.63)	409 (61.69)	32.884	<0.001
Marital status				
Spinsterhood	246 (57.21)	307 (69.93)	15.194	<0.001
Married	834 (54.58)	1,013 (66.86)	48.116	<0.001
Widowed	11 (57.89)	5 (26.32)	3.886	0.049
Divorced	27 (65.85)	17 (60.71)	0.190	0.663
Unstated marital status	7 (46.67)	23 (71.88)	2.811	0.094
Income (yuan/month)				
<1000	123 (41.84)	159 (58.89)	16.370	<0.001
1000~3000	327 (51.01)	275 (52.18)	0.158	0.691
3000~5000	522 (59.05)	683 (71.97)	33.924	<0.001
5000~10000	130 (69.52)	224 (85.82)	17.469	<0.001
≥10000	23 (85.19)	24 (92.31)	0.669	0.413
Payment method of medical expenses				
Basic medical insurance for urban workers	661 (64.55)	752 (76.66)	35.286	<0.001
Basic medical insurance for urban residents	188 (45.41)	254 (66.67)	36.313	<0.001
New rural cooperative medical insurance	217 (45.78)	283 (51.27)	3.074	0.080
Poverty relief	3 (100.00)	2 (33.33)	3.600	0.058
Commercial health insurance	10 (71.43)	6 (85.71)	0.525	0.469
Free medical care	12 (57.14)	12 (80.00)	2.057	0.151
Full medical care	16 (34.04)	30 (81.08)	18.490	<0.001
Else	18 (50.00)	26 (48.15)	0.030	0.863
Total	1,125 (55.34)	1,365 (67.14)	59.681	<0.001

### Answers to specific questions on hepatitis B prevention and treatment of respondents in the demonstration area

As shown in Table 3, the residents' awareness of the transmission route of hepatitis B was low: only 22.43%. After health education, it increased to 32.42% (*p*<0.001). Before and after health education, except the question: “What do you think is the main way to prevent hepatitis B infection at present?” There was no significant change in the correct answer rate, while the rest were improved (*p*<0.001).

**Table 3:** Analysis of knowledge, beliefs and practices regarding hepatitis B prevention of the respondents in the demonstration area

Topic	Number of correct answers (n,%)			
	Before health education	After health education	*X*^*2*^	*P*
Do you think hepatitis B is infectious?	1,589 (78.16)	1,792 (88.15)	7.153	0.007
Which of the following ways do you think hepatitis B can be transmitted? (Multiple choice)	456 (22.43)	659 (32.42)	50.923	<0.001
What do you think is the main way to prevent hepatitis B infection at present?	1,248 (61.39)	1,249 (61.44)	0.001	0.974
Do you know that the newborn should be vaccinated against hepatitis B within 24 hours after birth?	1,434 (70.54)	1,662 (81.75)	70.383	<0.001
Do you know after infection hepatitis b virus, although do not have special effect remedial method, but can still control illness after passing regular treatment?	1,485 (73.04)	1,671 (82.19)	48.980	<0.001
Have you been vaccinated against hepatitis B?	1,293 (63.60)	1,394 (68.57)	11.194	0.001
Are you willing to have your family vaccinated against hepatitis B?	1,905 (93.70)	1,968 (96.80)	21.590	<0.001

Table 4 shows that the health skill level of residents in the demonstration area was above 80% before health education and nearly 90% after health education (*P*<0.05).

**Table 4:** Hepatitis B health skills of respondents in the demonstration area

Topic	Number of correct answers (n,%)			
	Before health education	After health education	*X*^*2*^	*P*
Can you get the knowledge of Hepatitis B through the way such as getting online or consulting newspapers and periodicals?	1,683 (82.78)	1,799 (88.49)	26.906	<0.001
Can you read the instructions?	1,748 (85.98)	1,826 (89.82)	14.068	<0.001
Can you understand the hepatitis B popular science propaganda materials (such as pictorial, leaflet, pamphlet, etc.)?	1,744 (85.78)	1,801 (88.59)	7.153	0.007

### Effect evaluation of "Internet +" health education

#### Basic group information

The number of people who implemented health education and carried out the survey strictly by group is shown in Table 5, where the control group was traditional health education, intervention group 1 was "Internet +" health education, and intervention group 2 was traditional health education and "Internet +" health education. Before health education was carried out, the subjects were matched according to sex and age, and there was no statistical significance among the groups (*P*>0.05).

**Table 5:** Analysis of the groups of residents before different health education interventions

	Control group	Intervention group 1	Intervention group 2
Gender			
Male	195	181	190
Female	167	178	170
*X*^*2*^	0.898		
*P*	0.638		
Age (y)			
15~	45	48	46
25~	74	84	72
35~	74	70	60
45~	102	83	83
55~	67	74	99
*X*^*2*^	12.456		
*P*	0.132		

#### Effect evaluation of "Internet +" health education

**Fig. 1:**
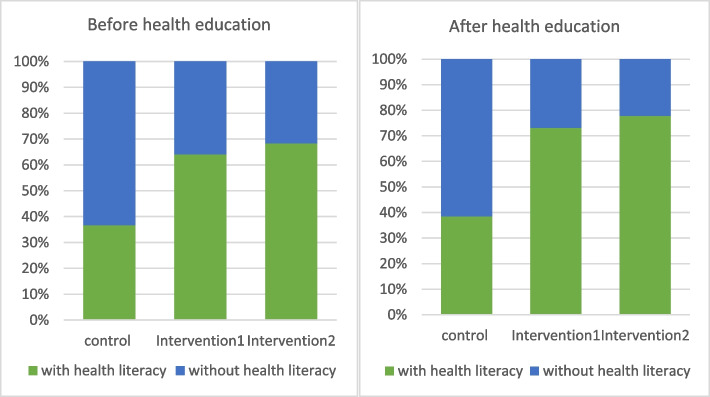
Comparison of the health literacy level of residents with different forms of health education after intervention

As shown in Table 6, the health literacy level of the residents in intervention group 1 and intervention group 2 was significantly higher than that before health education. Among them, the knowledge, belief, behaviour and health skills of the residents in intervention group 1 and intervention group 2 significantly improved. After health education, the health literacy level, concept and basic knowledge of hepatitis B, and attitude and behaviour of the residents towards hepatitis B (i.e., knowledge, belief and behaviour) significantly increased with statistical significance (*P*<0.05). The level of hepatitis B health literacy of residents in the intervention group was significantly higher than that in the control group, and the difference between groups was statistically significant (*P*<0.05). Further analysis showed that the health literacy level of residents in intervention group 1 was lower than that in intervention group 2. After health education, the health literacy level of residents in intervention group 1 and intervention group 2 was over 70%, and there was no statistical significance between the two groups (*P*>0.05) (Fig. 1).

**Table 6:** Evaluation of the intervention effects of different health education forms (n, %)

Group	Before health education	After health education	*X*^*2*^	*P*
Control group				
Knowledge-Attitude-Behaviour	79 (21.82)	109 (30.19)	6.582	0.010
Health skills	132 (36.46)	194 (53.74)	21.788	<0.001
Health literacy	133 (36.74)	139 (38.50)	0.240	0.624
Intervention group 1				
Knowledge-Attitude-Behaviour	163 (45.40)	206 (57.22)	10.050	0.002
Health skills	306 (85.24)	315 (87.50)	0.782	0.376
Health literacy	230 (64.07)	263 (73.06)	6.739	0.009
Intervention group 2				
Knowledge-Attitude-Behaviour	148 (41.11)	218 (61.24)	29.012	<0.001
Health skills	308( 85.56)	332 (93.26)	11.194	0.001
Health literacy	246 (68.33)	277 (77.81)	8.163	0.004
Total				
Knowledge-Attitude-Behaviour	390 (36.08)	533 (49.49)	39.645	<0.001
Health skills	844 (78.08)	841 (78.09)	0.000	0.995
Health literacy	609 (56.34)	679 (63.05)	10.091	0.001

## Discussion

The health literacy of infectious diseases from the perspective of public health includes three dimensions: knowledge, behaviour and skills related to infectious diseases, while a low level of health literacy is not conducive to the health of the population [6]. Research on the health literacy of infectious diseases has important significance in public health.

There was no statistical significance in gender or age in this study. The education level was mainly junior high school, senior high school and bachelor's degree or above. The ethnic group was mainly Han. Most of the participants were married. The main occupations were commercial and service workers, agricultural, forestry, fishery and water production personnel and professional and technical personnel. Medical expenses were mainly paid for by basic medical insurance for urban workers, basic medical insurance for urban residents and new rural cooperative medical care. Thus, the residents of the demonstration area were above average in cultural level and average in economic level.

Before health education, the health literacy level of residents' hepatitis B prevention and control basically showed a trend of decline with the increase of age, and the lowest in the age group of 55 + years old, which was basically consistent with other domestic research conclusions [7–12]. After health education, the level of health literacy of the elderly population over 55 years old still had a low level of health literacy. This may be related to poor understanding, inability to read traditional mission materials, inability to use mobile phones and other electronic devices to obtain mission materials, and physical conditions such as poor eyesight. Therefore, in view of the older people, especially the elderly over the age of 55, should look for more suitable health education, popularize knowledge about prevention and treatment of infectious diseases and health intervention, health communication and service quality of care for older people, so as to improve the prevention and treatment of infectious diseases health literacy level, at the same time also is of great significance to improve the quality of life of the elderly. The health literacy level of residents showed an increasing trend with the change of income, and the health literacy level of people with more than 10000 yuan/month was the highest, reaching more than 80%.This is consistent with other domestic studie s[10, 11]. before health education, the residents' awareness rate of hepatitis B transmission route was low, only 22.43%, which was consistent with the survey results of Liu Yang et al [12]. The awareness rate increased after health education. When asking “what is the most important way to prevent hepatitis B at present”, the correct answer rate before and after health education was about 61%, without significant statistical difference, which reminds us to emphasize the importance of hepatitis B vaccination in the process of health education in the future.

The form of "Internet +" health education may better improved the health literacy level of hepatitis B prevention and treatment of the residents. There were related studies at home and abroad, but few related to hepatitis B prevention and control [16–21]. The form of health education in the future can be combined with the new situation of social development to continuously innovate the health education mode. Since there were significant differences in health literacy levels of residents in each group before this analysis, the follow-up analysis should adjust the differences in age, gender and other aspects and make the evaluation indicators comparable before implementing intervention measures.

The purpose of health literacy surveys is to better target interventions. The fundamental purpose of health education intervention is to maximize the awareness of community residents regarding a specific health problem, improve individuals' decision-making ability in the process of prevention and disease care, and emphasize the cultivation of people's ability to recognize and use health information in daily life [19]. According to the KAP theory of health education, the awareness rate of hepatitis B prevention and treatment is closely related to its incidence [20]. The survey found that after the intervention of health education, the health literacy level of residents in the Zhejiang demonstration area for hepatitis B prevention and treatment reached 63.77%, which was higher than that of Chinese residents in 2020 (26.77%) [21]. However, the survey found that the awareness rate of transmission of hepatitis B was low. In the future, publicity and education in this area should be strengthened to enhance self-protection awareness and reduce the possibility of disease.

This study suggests that the health education model based on "Internet +" is feasible and efficient, but it also reminds us that the accessibility of these technologies to the elderly should be considered when exploring new health education models. Different methods should be adopted for different groups. For example, for elderly people, the publicity method with pictures and accompanying text can be adopted. International experience can be used for reference. The International Diabetes Federation (IDF) has launched a very practical health education tool called the "Conversation Map", which utilizes visual illustrations and activity cards for health education and has achieved good results [22].

In conclusion, the results of this survey indicated that community-based health promotion activities have improved residents' health literacy in hepatitis B prevention and treatment, and better results may be achieved by making full use of "Internet +" health education. However, there is still a large space to improve the health literacy level residents. In the future, targeted health education intervention can be carried out to explore new health education modes and effectively prevent and treat hepatitis B.

### Strengths and limitations

This study had 2 strengths. First, our study had an adequate sample size and a relatively long intervention period. Second, it verified the advantages of Internet-based health education and provided a basis for the prevention and treatment of infectious diseases in the future.

This study had the following limitations. First, the intervention was only conducted in rural area of Zhejiang Province, which may restrict the general applicability of our results. Second, Although the control group, intervention group 1 and intervention group 2 were set in the 6 demonstration areas at the beginning of the study, only 3 demonstration areas were given corresponding health education in strict accordance with the groups. Therefore, only three demonstration areas were analyzed in the Effect evaluation of "Internet +" health education.

## Data Availability

The datasets generated and/or analysed during the current study are not publicly available due to privacy or ethical restrictions. but are available from the corresponding author on reasonable request.
